# Neurotropism In Vitro and Mouse Models of Severe and Mild Infection with Clinical Strains of Enterovirus 71

**DOI:** 10.3390/v9110351

**Published:** 2017-11-20

**Authors:** Pin Yu, Linlin Bao, Lili Xu, Fengdi Li, Qi Lv, Wei Deng, Yanfeng Xu, Chuan Qin

**Affiliations:** Institute of Laboratory Animal Sciences, Chinese Academy of Medical Sciences (CAMS) & Comparative Medicine Center, Peking Union Medical College (PUMC); Key Laboratory of Human Diseases Comparative Medicine, Ministry of Health; Beijing Key Laboratory for Animal Models of Emerging and Reemerging Infectious Diseases, Beijing 100021, China; pinyucau@gmail.com (P.Y.); bllmsl@aliyun.com (L.B.); xull@cnilas.org (L.X.); icyli1019@hotmail.com (F.L.); qqmei-qiqi@163.com (Q.L.); dengwei717@163.com (W.D.); yanfxu@gmail.com (Y.X.)

**Keywords:** enterovirus 71, virulence, cytokine, chemokine, severe and mild mouse models

## Abstract

Enterovirus 71 (EV71) is a common etiological agent of hand, foot, and mouth disease and fatal neurological diseases in children. The neuropathogenicity of severe EV71 infection has been documented, but studies comparing mouse models of severe and mild EV71 infection are lacking. The aim of the study was to investigate the neurovirulence of EV71 strains and the differences in serum cytokine and chemokine levels in mouse models of severe and mild EV71 infection. Nine EV71 isolates belonging to the C4 subgenogroup (proposed as genotype D) displayed infectivity in human neuroblastoma SK-N-SH cells; moreover, ultrastructural observation confirmed viral particle replication. The survival rate of the severe model was 71.43% (5/7), and 60% (3/5) of the surviving severe model mice displayed sequelae of paralysis, whereas the only symptom in mild model mice was ruffled fur. Dynamic detection of serum cytokine and chemokine levels demonstrated that interleukin (IL)-5, IL-13, IL-6, monocyte chemotactic protein 1 (MCP-1), and chemokine (C-C motif) ligand 5 (also called Regulated upon Activation, Normal T-cell Expressed, and Secreted (CCL5/RANTES) were significantly up-regulated at the early period of infection, indicating that these factors might herald a severe outcome. Our findings suggest that elevated cytokines and chemokines may have potential value as prognostic markers in mouse models.

## 1. Introduction

Enterovirus 71 (EV71) is one of the viral causes of hand, foot, and mouth disease (HFMD), a common, mild, and self-limiting pediatric disease characterized by skin or mucosal vesicles or rash [1,2]. However, in young children, HFMD associated with EV71 infection can cause serious complications, such as acute flaccid paralysis, myocarditis, aseptic meningitis, brainstem encephalitis, neurogenic pulmonary edema, and even death [2,3]. EV71 belongs to the human-specific species Enterovirus A of *Picornaviridae*; this family also includes poliovirus, echovirus, and coxsackievirus [4]. The prototype EV71 strain BrCr was initially isolated from a patient with encephalitis who died in California, United States in 1969 [5,6]. Based on the structural VP1 gene, the EV71 viruses are originally categorized into three independent lineages, A, B, and C [7]. Genotype A comprises one member, the prototype BrCr strain. Genotypes B and C are each further classified into five subgenotypes, B1–B5 and C1–C5, respectively [8,9]. Since the 1980s, EV71 epidemics have occurred in Asian countries, including outbreaks in Malaysia in 1997 [10], Taiwan in 1998 [11], Singapore in 2000 [12], and Japan in 1997 and 2000 [13]. In China, EV71 was associated with large outbreaks in Shandong province in 2007 [14] and Fuyang in 2008 [15], and the incidence of HFMD is increasing annually. Recent EV71 epidemics in Hong Kong were attributed to subgenotype C4, which has been proposed to constitute a novel “double-recombinant” strains of “genotype D” [16,17].

Since it was first reported in California in 1969, EV71 has been recognized as the most vital neurotropic virus in most countries throughout the world, especially in the Asia-Pacific region [1,5,18,19]. Many patients with EV71 infection die from fatal pulmonary hemorrhage and edema [20]. However, autopsy studies reveal that EV71 also causes neural disorders by inducing an inflammatory response in the CNS and that cardiopulmonary disease and complications are neurogenic [3,21,22]. Therefore, understanding the neuropathogenesis and neurotropism of fatal EV71 infection is of substantial importance.

A reliable animal model that mimics severe and mild EV71 infection is essential for understanding clinical prognosis. Previous studies of experimental EV71 infection have explored neonatal mouse [23,24,25,26,27], monkey [28], and gerbil [29] animal models. Both mouse-adapted and non-mouse-adapted EV71 strains infect neonatal mice, causing massive lesions of limb muscles, the brainstem, and anterior horn areas. These mice exhibit neuronal necrosis and apoptosis in the brainstems and the anterior horn areas of the spinal cord, suggesting the neurotropism of EV71 infection [23,25]. Transgenic mice expressing the human scavenger receptor class B, member 2 (hSCARB2), which functions as a receptor for EV71, are susceptible to EV71 infection and exhibit ataxia, paralysis, and death [26,27]. However, these mouse models differ significantly from the human disease because most human patients exhibit mild infection, while a minority of patients develop severe complications [30]. Moreover, there has not been any progress in the elucidation of the pathogenesis of fatal EV71 infection, because appropriate animal models of severe and mild infection have not yet been established. Consequently, suitable animal models of severe and mild EV71 infection would provide unique tools for studying fatal EV71 pathogenesis. However, much research has focused on understanding the neuropathogenesis of the EV71 mouse model, whereas immunopathogenesis aspects and serological comparisons of mouse models of severe and mild EV71 infection have been largely neglected. High levels of interleukin-1(IL-1), IL-6, IL-10, IL-13, gamma interferon (IFN-γ), and tumor necrosis factor alpha (TNF-α) have been detected in the sera and cerebral spinal fluid of EV71-infected patients [31,32,33]. Moreover, levels of IL-1, IL-6, and TNF-α in the cerebral spinal fluid are significantly elevated in patients with neurogenic pulmonary edema and encephalitis. Therefore, it has been proved that the massive proinflammatory cytokine and chemokine induction is responsible for the neuropathogenesis and clinical severity of EV71 infection [32,33,34,35,36].

Herein, we investigate the in vitro neuropathogenesis of EV71 strains in human and mouse neuroblastoma cells; moreover, we compare pathological characteristics, viral replication in tissues, and serum proinflammatory cytokine and chemokine levels in mouse models of severe and mild EV71 infection. The complete genomes of nine EV71 strains isolated from patients from Hunan, Ningxia, and Guangdong in China in 2010–2012 with mild, severe, and fatal infection were sequenced, and their virulence and neurotropism in vitro were analyzed. To get further insight into the neuropathogenesis of severe EV71 infection, the pathological changes, viral distribution sites, tissue viral loads, and serum cytokine and chemokine levels were determined for the severe and mild mouse models.

## 2. Materials and Methods

### 2.1. Ethics Statement

This research on EV71 was discussed among the staff members of the Department of Pathogen Biology at the Institute of Laboratory Animal Science of the Chinese Academy of Medical Sciences and Peking Union Medical College. The experiments and protocols for animal models of EV71 infection were discussed explicitly and extensively among the staff members of the Department of Pathogen Biology. The experimental protocol was approved by the Animal Care and Use Committee of the Institute of Laboratory Animal Science of the Chinese Academy of Medical Sciences. These discussions were followed by consultations with biosafety officers and facility managers at the Institute of Laboratory Animal Science of Peking Union Medical College. The approved registration number for this study is ILAS-PC-2012-001. All research procedures were approved by the Institute of Laboratory Animal Science (ILAS) Institutional Animal Care and Use Committee and Laboratory Safety Committee (LSC). The methods were carried out in accordance with the animal model experiments and protocols of ILAS Institutional Animal Care and Use Committee and LSC.

### 2.2. Cells and Viruses

RD (rhabdomyosarcoma) cells were maintained in Dulbecco’s modified Eagle’s medium (DMEM) containing 10% fetal bovine serum (FBS) with 50 U/mL of a penicillin/streptomycin mixture and incubated at 37 °C in a humidified 5% CO_2_ atmosphere. Human neuroblastoma SK-N-SH cells were maintained in DMEM with 50 U/mL of a penicillin/streptomycin mixture and 10% FBS and incubated at 37 °C in a 5% CO_2_ atmosphere. Mouse neuroblastoma Neuro-2a (N2a) cells were maintained in minimum essential medium (MEM) supplemented with 50 U/mL of penicillin/streptomycin and 10% FBS at 37 °C in a 5% CO_2_ atmosphere.

### 2.3. Virus Infection and Sample Collection

Nine strains of EV71 were grown individually in RD cells. All EV71 strains belonged to subgenogroup C4 (proposed as genotype D) and were originally isolated from mild, severe, and fatal clinical cases during HFMD outbreaks in different regions (Table S1). To prepare viral stocks, viruses were propagated for one more passage in RD cells as reported previously [37]. Working virus stocks contained a 10^4.5^ 50% tissue culture infective dose (TCID50) per mL.

In vitro neuroinfection studies were conducted using low-serum (2%) DMEM for SK-N-SH cells and low-serum (2%) MEM for N2a cells. The cells were seeded in 24-well plates (Corning, Lowell, MA, USA) at a density of 1 × 10^5^ cells per well in 1 mL of culture medium, and the cells in each well were infected with 100 TCID50 of one of the nine EV71 strains. Culture medium served as the control in each experiment. The cell supernatant was harvested at 2, 6, 12, 24, 48, and 72 h post-infection (hpi) for viral RNA extraction and quantitative detection, and cell climbing slices were collected at 12 and 24 hpi for double-label immunofluorescence analysis.

To obtain infected SK-N-SH cells for transmission electron microscopy (TEM) observation, cells were digested in a cell suspension by 0.25% Trypsin-EDTA (ethylenediaminetetraacetic acid) and centrifuged at 5000 rpm for 10 min. The resulting cell pellet was then fixed in glutaraldehyde fixative for ultrathin section preparation and observation by TEM (JEM-1400).

### 2.4. Double-Label Immunofluorescence Assay

EV71-infected and non-infected SK-N-SH and N2a cells on cell-climbing slices were fixed with 4% paraformaldehyde at room temperature for 2 h and permeabilized with 0.5% Triton X-100 on ice for 10 min, followed by 3 washes with phosphate-buffered saline (PBS). The slices were then blocked with 5% bovine serum albumin at 37 °C for 30 min. The slices were incubated with anti-EV71 mouse monoclonal antibody (MAB979, Millipore, California, USA) diluted 1:500 in PBS and anti- neuron-specific enolase (NSE) rabbit monoclonal antibody (ab79757, Abcam, Cambridge, Massachusetts, United States of America) diluted 1:200 in PBS at 4 °C overnight. After 3 washes with PBS, the cells were incubated with fluorescein isothiocyanate (FITC)-conjugated anti-rabbit antibody and tetramethylrhodamine isothiocyanate (TRITC)-conjugated anti-mouse antibody at a 1:200 dilution at 37 °C for 1 h in the dark. The cells were washed 3 times with PBS and incubated with 1 μg/mL 4,6-diamidino-2-phenylindole (DAPI) for 5 min. After 3 washes with PBS, the cells were covered and observed under an Olympus Confocal Laser Scanning Microscope.

### 2.5. RNA Extraction and Reverse Transcription Polymerase Chain Reaction 

Viral RNA of the nine clinical EV71 strains was extracted from cell supernatants using an RNeasy Mini Kit (Qiagen, Hilden, Germany). Reverse transcription (RT) reactions were performed using the Superscript III First Strand Synthesis Kit (Invitrogen, Carlsbad, United States of America ) according to the supplier’s instructions. Nine pairs of overlapping primers (Table S2) were designed based on the conserved regions of EV71. The polymerase chain reaction (PCR) parameters for all primer pairs were as follows: complementary DNA (cDNA) was denatured at 94 °C for 5 min. Amplification was performed using KOD-plus-DNA Polymerase (Toyobo, Tokyo, Japan) with 35 cycles of denaturation for 30 s at 94 °C, primer annealing for 45 s at 56 °C, and elongation for 1 min at 68 °C, followed by extension at 68 °C for 10 min. The reactions were analyzed by electrophoresis in 1.0% agarose gels.

### 2.6. Nucleotide Sequencing

Amplicons were either sequenced directly or purified with an E.Z.N.A. Gel Extraction Kit (OMEGA), cloned into the pGEM-T Easy vector (Promega, Madison, United States of America) and sequenced with T7 and SP6 primers. All sequencing was performed by Life Technologies (Shanghai, China). The EV71 full-length genomes were acquired by assembling the fragments using the DNAMAN 5.0 software.

### 2.7. Phylogenetic Analysis

Multiple alignments of the nine clinical EV71 strains were constructed using the Clustal W multiple alignment program of the software BioEdit (version 7.0.5.2). Phylogenetic and molecular evolutionary analyses were conducted using the neighbor-joining method using the MEGA 4.0 software with 1000 bootstrap replications [38].

### 2.8. Nucleotide Sequence Accession Number

The complete genome sequences of the nine EV71 strains (Table S1) have been deposited in the GenBank database under accession numbers KF004552 through KF004560.

### 2.9. Severe and Mild EV71 Mouse Models and Sample Collection

Severe and mild EV71 mouse models were established as follows. Briefly, BALB/c mice at nine days of age (n = 90) were fasted for 4 h and inoculated intraperitoneally (i.p.) with 10^4.5^ TCID50 of the severe clinical strain of EV71 NX10-36 (GenBank accession number KJ004557) or the mild clinical strain NX10-147 (GenBank accession number KJ004556) in 250 μL. The control mice (n = 12) were injected with the same volume of RD cell supernatants, and 3 mice were sacrificed to extract control serum. Body weights and clinical signs were monitored daily up to 14 days post-infection (dpi). Clinical symptoms were scored as follows: 0, healthy; 1, ruffled fur and hunchbacked appearance; 2, weakness in the hind limbs; 3, paralysis in a single hind limb; 4, paralysis in both hind limbs; 5, moribundity and death. Three mice per infected group were sacrificed for serum extraction at 3, 6, and 12 hpi and 1, 2, 3, 5, 7, and 9 dpi. In addition, 3 mice per group were sacrificed on 3 and 5 dpi, and the brainstem, spinal cord, lung, limb muscle, and jejunum tissues were immediately collected and fixed in 2.5% (vol/vol) glutaraldehyde-polyoxymethylene mixture fixative for histopathological and immunohistochemical examinations, respectively. Three mice per group were also sacrificed to collect tissues for viral RNA extraction. 

### 2.10. RNA Extraction and Quantitative Detection of Viral RNA by Reverse Transcription Polymerase Chain Reaction

Total RNA was extracted from different tissues and cell supernatants using an RNeasy Mini kit (Qiagen, Hilden, Germany) according to the manufacturers’ instructions. RNA was reverse transcribed into cDNA using the Superscript III First Strand Synthesis Kit (Invitrogen, Carlsbad, United States of America). The viral load in different tissues was determined using SYBR Green PCR Master Mix with the primers EV71-S (5′-GCAGCCCAAAAGAACTTCAC-3′) and EV71-A (5′-ATTTCAGCAGCTTGGAGTGC-3′) for nucleotides 2366–2592 of EV71/Queenmary/HongKong/2012. Quantitative RT-PCR was performed using a StepOne PCR system (ABI, Carlsbad, United States of America). PCR amplifications were performed in duplicate at 94 °C for 3 min followed by 35 cycles of 94 °C for 30 s, 60 °C for 30 s, and 72 °C for 30 s. Each assay was performed in triplicate, and a standard curve was constructed using serial 10-fold dilutions of the stock EV71 strain (GenBank accession number KF444809, 10^5.0^ TCID50/mL).

### 2.11. Histopathology Examination and Immunohistochemistry

The fixed samples were dehydrated and embedded in paraffin according to standard procedures, and 4 μm sections were prepared with a microtome. Some sections were stained with hematoxylin-eosin (HE) by routine methods to observe histological lesions. For Nissl staining, histological sections of the brainstems and spinal cords were dewaxed, rehydrated and stained with 1% toluidine blue, dehydrated through graded alcohols (70, 80, 95, 100% 2 × ), placed in xylene, and mounted with neutral gum. Immunohistochemistry (IHC) was performed using a standard avidin-biotin immunoperoxidase technique [39]. An anti-EV71 rabbit polyclonal antibody (GTX124261, GeneTex, Irvine, California, United States of America) was used as the primary antibody for IHC. Sections for the negative control were incubated with non-immune serum. The histological changes and intensity of EV71 antigen labeling were observed under a microscope by two independent pathologists.

### 2.12. Cytometric Bead Array (CBA)

Prior to the commencement of the cytometric bead array (CBA), serum was collected from mixed blood of mice in each group, in consideration of the insufficiency of one suckling mouse’s blood collection for the test. Cytokines and chemokines in individual serum samples were quantified by flow cytometry (FACSCalibur, BD Biosciences, San José, California, United States of America ) using the CBA Inflammatory Kit (BD Bioscience). IL-1β, IL-2, IL-4, IL-5, IL-6, IL-10, IL-13, IFN-γ, TNF-α, chemokine (C-C motif) ligand 5 (also called Regulated upon Activation, Normal T-cell Expressed, and Secreted, CCL5/RANTES), and monocyte chemotactic protein 1 (MCP-1) were measured using the mouse inflammation cytokine CBA detection system (BD Biosciences) according to the manufacturer's instructions.

### 2.13. Statistical Analysis

A two-tailed Student’s *t*-test was used to determine significant differences. Analyses were performed using Prism Software version 5.0 for Windows (GraphPad, San Diego, CA, USA). The data shown are the mean of three independent experiments ± SD. For all statistical tests, *p* < 0.05 was considered significant.

## 3. Results

### 3.1. Virulence and Neurotropism of EV71 In Vitro

**Figure 1:**
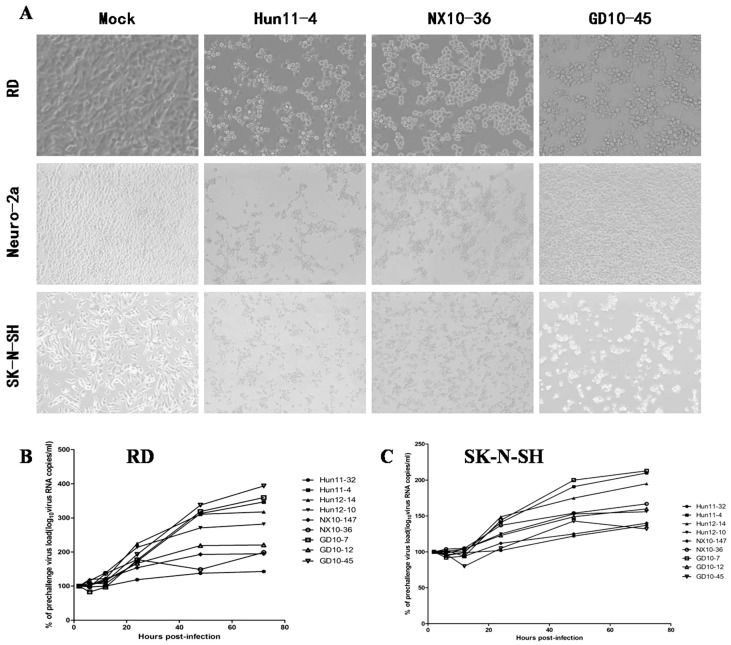
Enterovirus 71 (EV71) strain infection and replication in rhabdomyosarcoma (RD) cells, mouse neuroblastoma Neuro-2a (N2a) cells and human neuroblastoma (SK-N-SH) cells. (**A**) Images of RD, N2a, and SK-N-SH cells acquired 24 hours post infection (hpi) after infection with 100 50% tissue culture infective dose (TCID50) of three representative EV71 strains, including clinical mild strain Hun11-4, clinical severe strain NX10-36, and clinical fatal strain GD10-45 (magnification 200×). (**B**) Replicative curve of EV71 in RD cells. Total RNA was prepared from infected and uninfected RD cells at 2, 6, 12, 24, 48, and 72 hpi. After reverse transcription, VP-1 specific primers were used to amplify the viral VP1 gene transcript from the complementary DNA (cDNA), confirming viral replication in infected RD cells. (**C**) Replicative curve of EV71 in SK-N-SH cells. Total RNA was prepared from infected and uninfected SK-N-SH cells at 2, 6, 12, 24, 48, and 72 hpi. After reverse transcription, VP-1 specific primers were used to amplify the viral VP1 gene transcript from the cDNA, confirming viral replication in infected SK-N-SH cells.

**Figure 2:**
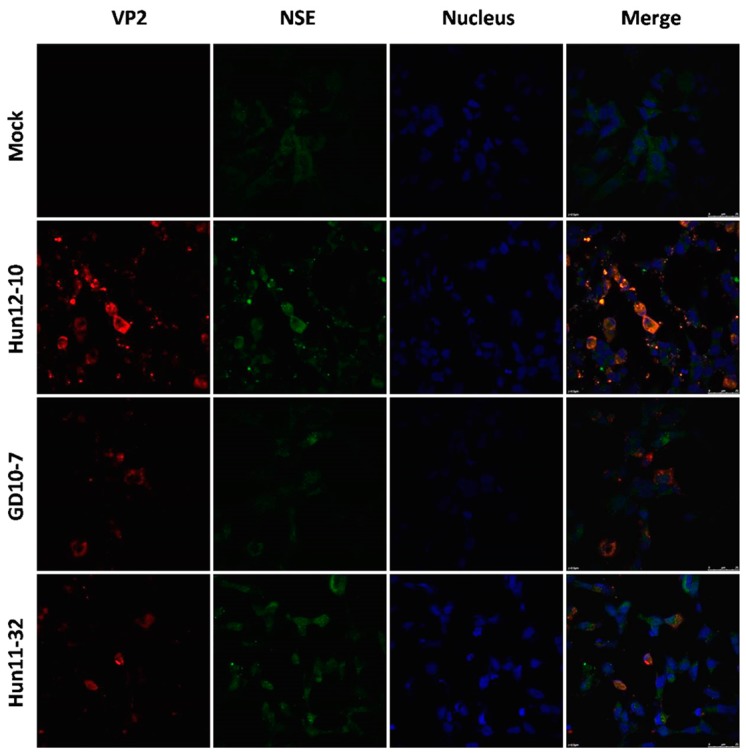
Immunofluorescence assay in human neuroblastoma SK-N-SH cells (magnification 630×). Cells were infected with EV71 Hun12-10, GD10-7, or Hun11-32. Mock-infected and infected cells were fixed at 24 hpi and incubated with mouse anti-EV71 VP2 antibody and rabbit anti- neuron-specific enolase (NSE) antibody, followed by incubation with fluorescein isothiocyanate (FITC)-(green) and tetramethylrhodamine isothiocyanate (TRITC)-(red) conjugated secondary antibodies. The cells were also stained with 4,6-diamidino-2-phenylindole (DAPI) (blue) to visualize the nuclei.

**Figure 3:**
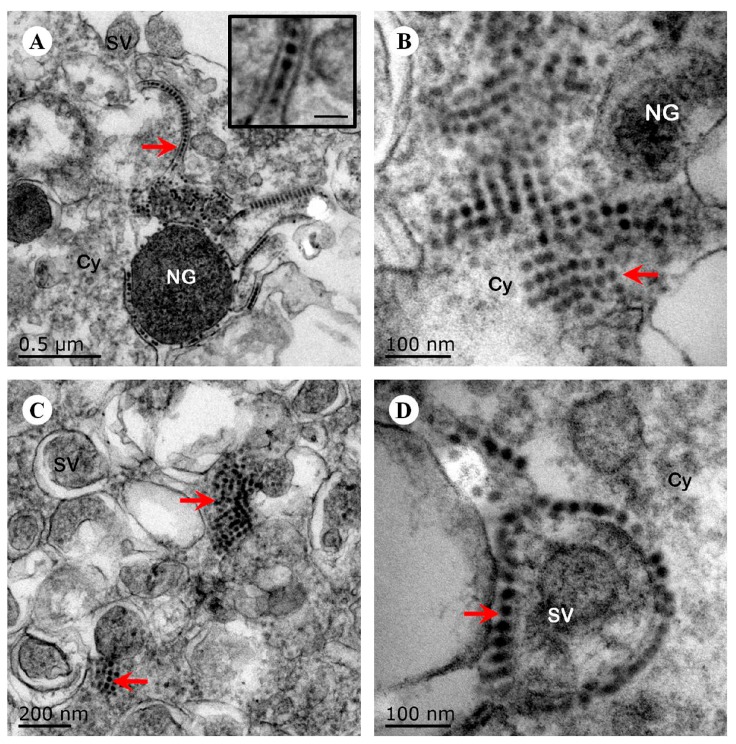
Ultrastructural observation of human neuroblastoma SK-N-SH cells infected with EV71 Hun11-32. (**A**) The viral particles (red arrow) are located in a line between the membranes (scale bar, inset, 50 nm). (**B**) The viral particles (arrow) clustered as crystals in the cytoplasm. (**C**) The viral particles (arrow) clustered as crystals next to the secretory vesicles in the cytoplasm. (**D**) The viral particles (arrow) surrounded the membrane of the secretory vesicles. NG, neurosecretory granules; SV, secretory vesicles; Cy, cytoplasm.

**Figure 4:**
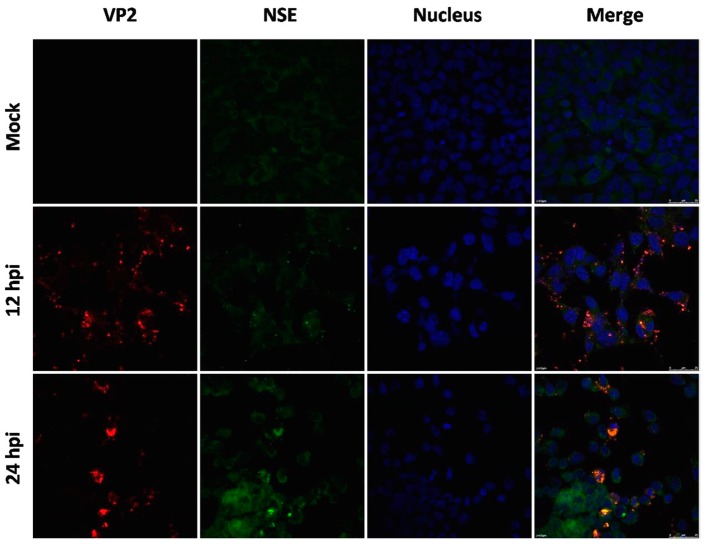
Immunofluorescence assay in mouse neuroblastoma Neuro-2a cells (magnification 630 ×). Cells were infected with EV71 Hun11-4, and mock-infected or infected cells were fixed at 12 and 24 hpi and incubated with mouse anti-EV71 VP2 antibody and rabbit anti-NSE antibody, followed by incubation with FITC-(green) and TRITC-(red) conjugated secondary antibodies. The cells were also stained with DAPI (blue) to visualize the nuclei.

To examine hypervirulence and neurotropism in vitro, we inoculated human neuroblastoma SK-N-SH cells, mouse neuroblastoma N2a cells, and RD cells with the nine clinical EV71 strains, and the cells were examined at certain time points. All nine strains infected the SK-N-SH and RD cells. As shown in Figure 1A, a significant cytopathic effect (CPE) was observed in the EV71-infected RD and SK-N-SH cells at 24 hpi, and no CPE was observed in uninfected cultures. However, only EV71 strains Hun11-4 and NX10-36 produced significant CPEs in N2a cells. We examined the expression levels of the EV71 VP1 gene by real-time RT-PCR using VP1-specific primers, which confirmed that these nine clinical EV71 strains replicated in RD cells (Figure 1B). Investigation of EV71 infection in SK-N-SH and N2a cells revealed a significant increase in the viral loads of these nine strains in human neural SK-N-SH cell supernatants (Figure 1C). However, slight EV71 propagation and CPEs were detected in mouse neural N2a cells for only two of the nine clinical strains (data not shown). In non-infected cells, no VP1 transcript was detected. For further proof of the replication and propagation of EV71 in human and mouse neural cells, we performed double-label immunofluorescence analysis to visualize the EV71 antigen in neural cells. As shown in Figure 2, viral proteins were detected in SK-N-SH cells at 24 hpi, and ultrastructural observation revealed that viral particles were replicated between the membranes (Figure 3A) and clustered as crystals in the cytoplasm (Figure 3B). Viral particles that appeared to be secreted from SK-N-SH cells were also adjacent and proximal to secretory vesicles (Figure 3C,D). In N2a cells, EV71 Hun11-4 strain was detected at 12 and 24 hpi, as shown in Figure 4, but was not significantly increased. Thus, our data show that the EV71 virus can infect and propagate in human neural SK-N-SH cells, while some of the EV71 strains were unable to infect and replicate mouse neural N2a cells.

### 3.2. Phylogenetic Relationships of EV71 Strains

**Figure 5:**
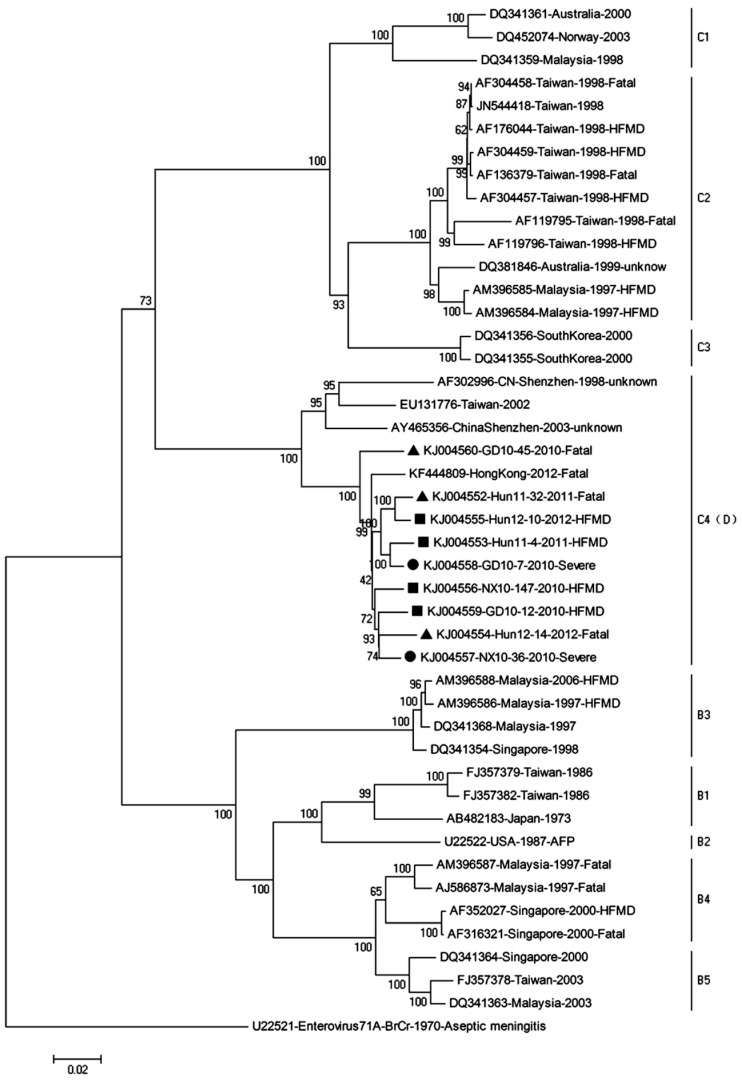
Phylogenetic analysis based on the complete genomes of the EV71 strains. Phylogenetic trees were generated by the neighbor-joining method with 1000 bootstraps from representative strains of known subgenotypes. The ▲ icon indicates fatal cases; ● indicates severe cases; ■ indicates mild hand, foot, and mouth disease (HFMD) cases. KJ004556 (NX10-147) and KJ004557 (NX10-36) were used to establish the mouse models of severe and mild EV71 infection.

This study included complete genome sequencing and phylogenetic analysis of nine EV71 strains isolated from EV71 patients of outbreaks in 2010, 2011, and 2012 in Hunan, Ningxia, and Guangdong province, respectively. The four strains classified as mildly virulent strains (Hun11-4, Hun12-10, NX10-147, GD10-12) were obtained from patients of mild symptoms, which are herpangina, acute pharyngitis, or HFMD, whereas the two strains defined as severely virulent strains (NX10-36, GD10-7) were isolated from patients with central nervous system (CNS) involvement; the three strains defined as fatal virulent strains (Hun11-32, Hun12-14, GD10-45) were derived from patients with fatal outcomes. The GenBank accession number, clinical characteristics, region source, year of isolation, and subgenotype of each of the EV71 isolates included in the study are provided in Table S1. To determine the genetic characteristics of mild, severe, and fatal virulent strains, the nine EV71 complete genome sequences of this study and the sequences of reference strains available in GenBank were subjected to phylogenetic analysis (Figure 5). The results of this analysis demonstrated that all of the mild, severe, and fatal virulent EV71 isolates isolated in China in 2010–2012 that were included in this study belong to subgenogroup C4, which has been proposed as genotype D.

### 3.3. Establishment of Mouse Models of Severe and Mild EV71 Infection

**Figure 6:**
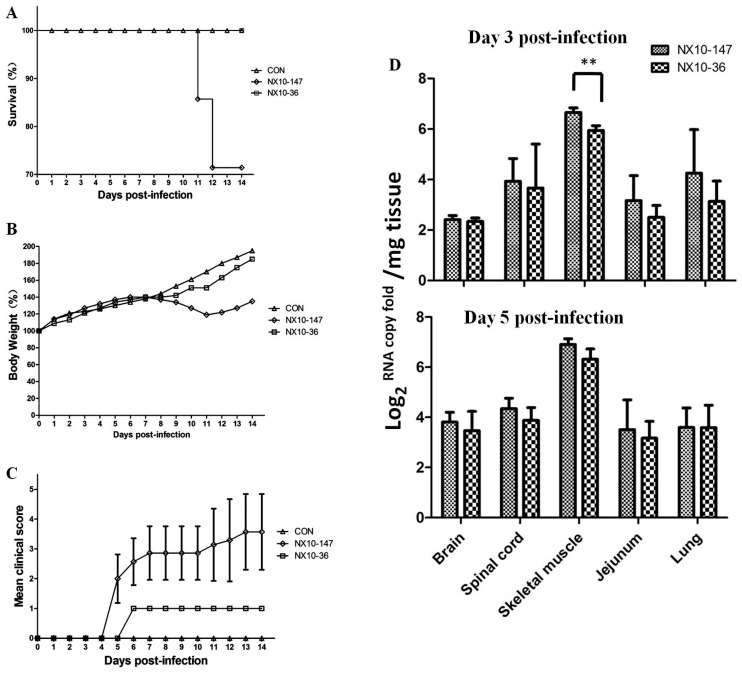
Survival rate, body weight, clinical score, and virus replication in various mouse tissues after the intraperitoneal (i.p.) inoculation challenge with 10^4.5^ TCID50 of EV71 NX10-147 and NX10-36 as well as a mock control. Survival rates (**A**), body weight (**B**), and clinical scores (**C**) were monitored daily after challenge. The viral load (**D**) was calculated from a standard curve of quantitative real-time RT-PCR results obtained with 10-fold serial dilutions of the KF444809 virus strain. The data shown here are the mean viral loads ± standard errors (3 mice each). The difference in the viral loads in the skeletal muscle at 3 days post-infection (dpi) were statistically significant by Student’s *t*-test (** p < 0.01).

In the present study, we determined that i.p. inoculation of 10^4.5^ TCID50 EV71 clinical strains (NX10-147 and NX10-36) in mice at nine days of age resulted in severe and mild clinical manifestations. Mice inoculated with NX10-147 developed hind limb paralysis induced by EV71 infection from 6 dpi and even death from 11 dpi, whereas mice inoculated with NX10-36 exhibited no sign of disease and developed ruffled fur and a hunchbacked appearance at 6 dpi. To further evaluate the severe and mild EV71 mouse models in detail, survival rates, body weight, and clinical scores were measured (Figure 6A–C). At 5 dpi, 2/7 (28.57%) mice of the severe model inoculated with NX10-147 developed hind limb paralysis. At 7 dpi, 4/7 (57.14%) mice of the severe model exhibited paralysis, and body weight began to decrease. The mice of the severe model died at 11–12 dpi, and the survival rate was 71.43% (5/7). Moreover, 60% (3/5) of the surviving mice of the severe model had sequelae of paralysis. However, the mice of the mild model inoculated with NX10-36 exhibited ruffled fur at 6 dpi, and all survived to 14 dpi. No difference in body weight between the mice of the mild model and those of the control group was found.

### 3.4. EV71 Replication in Severe and Mild EV71 Mouse Models

To further investigate the replication of EV71 in the severe and mild mouse models, tissue samples, including brain, spinal cord, skeletal muscle, jejunum, and lung, were collected from EV71-infected mice 3 and 5 dpi with 10^4.5^ TCID50 of EV71 strains (NX10-147 and NX10-36) (Figure 6D), and viral loads were determined. At 3 and 5 dpi, EV71 was detected in the brain, spinal cord, skeletal muscle, jejunum, and lung. High viral loads (10^6.0^ to 10^7.0^ copies/mg) were detected in the skeletal muscle of the severe and mild mouse models at 3 dpi. However, at 5 dpi, the viral loads did not substantially increase but were significantly different in the muscles of the severe and mild mouse models (*p* < 0.01). A low virus titer (10^2.0^ copies/mg) was detected in the brain at 3 dpi and gradually increased to 10^4.0^ copies/mg at 5 dpi, demonstrating that EV71 was transmitted to the CNS as the infection progressed. The virus was detected in tissue samples from spinal cord, jejunum, and lung, with viral loads from 10^3.0^ to 10^4.0^ copies/mg at 3 dpi; these medium viral loads were maintained at 5 dpi. The skeletal muscle and spinal cord maintained high viral loads, demonstrating that these organs were sites of ongoing viral replication and transmission to the brain.

### 3.5. Pathology in Severe and Mild EV71 Mouse Models

Histopathological and immunohistochemical examination revealed typical lesions and positive EV71 antigens in various tissues in the severe and mild mouse models after i.p. inoculation with a dose of 10^4.5^ TCID50 of EV71 strains (NX10-147 and NX10-36). No obvious lesions were observed in the control group, and the organs were negative for EV71 antigen.

**Figure 7:**
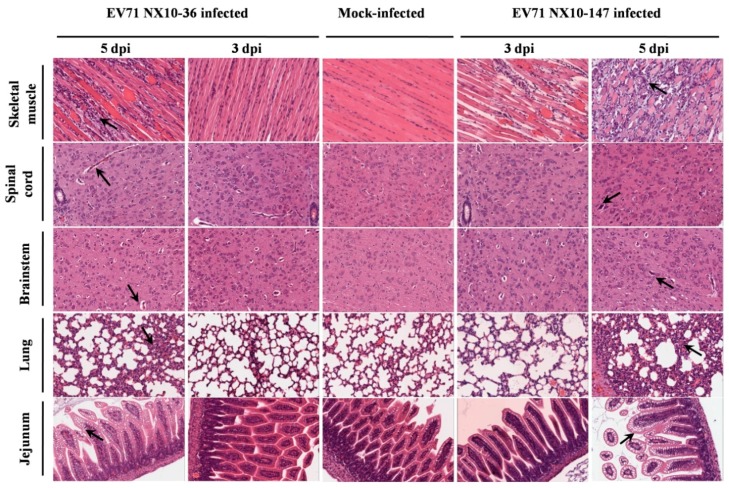
Histological analysis of various mouse tissues. BALB/c mice at nine days of age were inoculated intraperitoneally with 10^4.5^ TCID50 of EV71 NX10-147 or NX10-36, and the mice were sacrificed at the indicated time points (3 mice each). Tissues were removed, and tissue sections were subjected to hematoxylin-eosin staining, followed by histopathological examination by light microscopy. The data shown are representative images of each group of mice at 3 and 5 dpi (magnification 200×). The arrows indicate the typical characteristics of the lesions in different tissues at 5 dpi. There were no obvious pathological features in these tissues in the control group.

**Figure 8:**
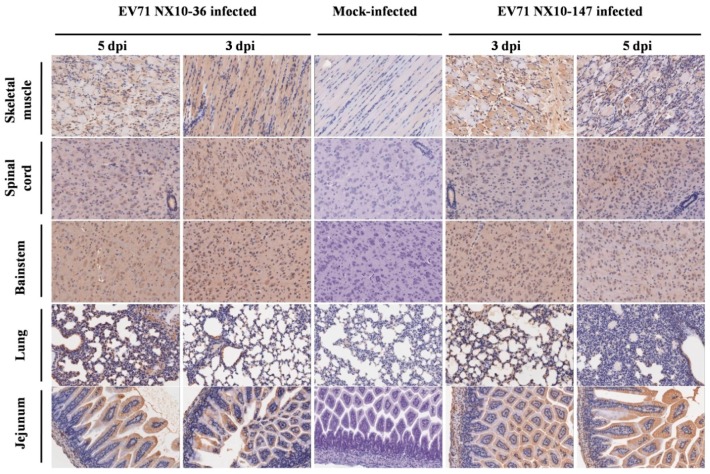
Immunohistochemical analysis of tissues from EV71-infected mice. Nine-day-old mice were inoculated i.p. with 10^4.5^ TCID50 of EV71 NX10-147 or NX10-36. The sections were positive (brown particles) for EV71 antigen in the skeletal muscle, spinal cord, brainstem, lung, and jejunum (magnification 200×). No positive EV71 antigen staining was observed in the tissues of mock controls.

In the severe EV71 mouse model, hind limb paralysis appeared at 5 dpi. The skeletal muscle exhibited severe necrotic myositis with massive inflammatory cell infiltration at 3 and 5 dpi. At the same time points, the spinal cords and brainstems revealed different degrees of lesions. Neuronal swelling and edema were observed at 3 dpi. At 5 dpi, focal neuronal death and neuronophagia were detected in spinal cords, and different degrees of degeneration and necrosis as well as the disappearance of Nissl bodies were observed in the brainstems and spinal cords (Figure S1). Localized emphysema was observed in the lungs at 3 and 5 dpi, and the lungs exhibited massive interstitial pneumonia with inflammatory infiltration and congestion in the capillary vessels of alveolar septa. The jejuna revealed mild and massive vacuolar degeneration in mucosal epithelial cells at 3 and 5 dpi, respectively (Figure 7). Furthermore, the EV71 antigen was detected in these tissues by immunohistochemical analysis. At 3 and 5 dpi, the spinal cords, brainstems, jejuna, and skeletal muscles exhibited intense staining of the EV71 antigen, demonstrating active virus replication in these organs, while the lungs displayed moderate staining for the EV71 antigen at 5 dpi (Figure 8).

By contrast, no severe clinical manifestations were observed in the mild mouse model. Microscopically, at 3 and 5 dpi, the skeletal muscles indicated myositis with inflammatory infiltration; however, no massive necrosis was observed in the myocytes. In the brainstems and spinal cords, degeneration in the neurons and edema was observed, and disappearance of Nissl bodies was observed in the spinal cords at 5 dpi. No obvious lesions were observed in the lungs and jejuna at 3 dpi. However, at 5 dpi, focal interstitial pneumonia was identified in the lungs, and the jejuna revealed a high degree of vacuolar degeneration in epithelial cells (Figure 7). As in the severe mouse model, the EV71 antigen was found in these tissues. At 3 and 5 dpi, the spinal cords, brainstems, and skeletal muscles displayed intense staining of the EV71 antigen, and the lungs and jejuna exhibited mild or moderate staining of the EV71 antigen at 3 and 5 dpi (Figure 8).

### 3.6. Detection of Cytokines and Chemokines in the Sera of Severe and Mild EV71 Mouse Models

**Figure 9:**
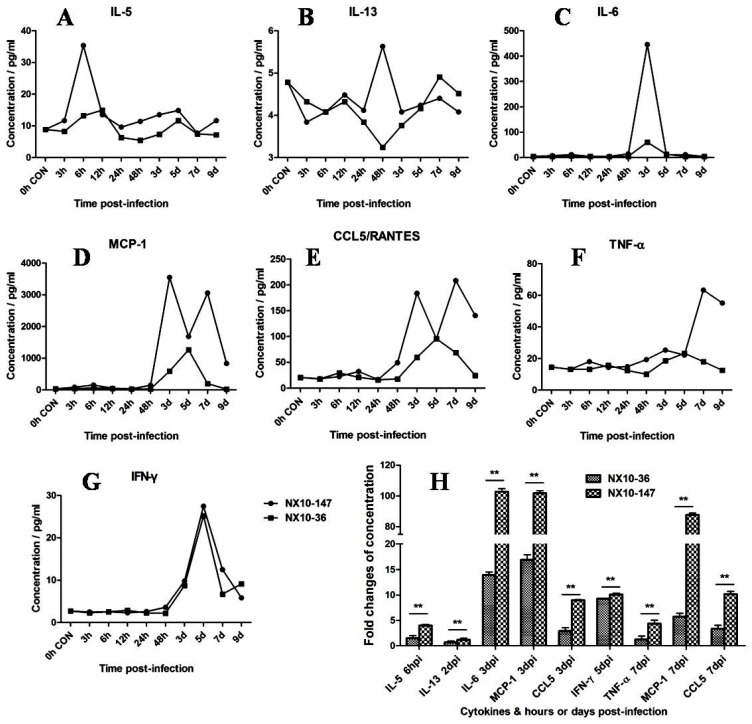
Dynamic detection of inflammatory cytokines and chemokines in the sera of EV71 (NX10-147 and NX10-36)-infected mice sacrificed at 3, 6, and 12 hpi and 1, 2, 3, 5, 7, and 9 dpi. The mean serum concentrations of cytokines and chemokines (**A**) interleukin (IL)-5, (**B**) IL-13, (**C**) IL-6, (**D**) monocyte chemotactic protein 1 (MCP-1), (**E**) chemokine (C-C motif) ligand 5 (also called Regulated upon Activation, Normal T-cell Expressed, and Secreted) (CCL5/RANTES), (**F**) tumor necrosis factor (TNF)-α, and (**G**) interferon (IFN)-γ are shown. The fold changes in IL-5(6 hpi), IL-13(2 dpi), IL-6(3 dpi), MCP-1(3 and 7 dpi), CCL5/RANTES (3 and 7 dpi), IFN-γ (5 dpi), and TNF-α (7 dpi) in the mouse models of severe and mild infection are shown (**H**) (** p < 0.01).

We investigated the cytokine and chemokine induction profile induced by EV71 infection in the severe and mild mouse models. As shown in Figure 9A–G, dynamic detection of inflammatory cytokines and chemokines in the sera of mice with severe and mild infection sacrificed at 3, 6, and 12 hpi and at 1, 2, 3, 5, 7, and 9 dpi was accomplished by CBA detection. In the severe model, a serum cytokine storm was detected from 6 hpi to 7 dpi, while a modest induction of cytokines and chemokines was observed in the mild model. Compared to the mild mouse model, IL-5 (6 hpi), IL-13 (2 dpi), IL-6 (3 dpi), MCP-1 (3 and 7 dpi), CCL5/RANTES (3 and 7 dpi), IFN-γ (5 dpi), and TNF-α (7 dpi) were all significantly increased in the severe mouse model (Figure 9H) (*p* < 0.01). IL-5 was up-regulated up to 4-fold at 6 hpi in the severe model, whereas IL-5 was increased marginally 1.5-fold in the mild model. At 2 dpi, IL-13 was mildly up-regulated as much as 1.2-fold in the severe model but was down-regulated to 0.7-fold in the mild model. The levels of IL-6 and MCP-1 increased by more than 100-fold in the severe model at 3 dpi and approximately 14- and 17-fold, respectively, in the mild model. CCL5/RANTES increased 9- and 10-fold at 3 and 7 dpi, respectively, in the severe model. By contrast, CCL5/RANTES was up-regulated 2.9- and 3.3-fold at 3 and 7 dpi, respectively, in the mild model. The levels of IFN-γ at 5 dpi increased by approximately 10- and 9.2-fold in the severe and mild models, respectively. The levels of TNF-α and MCP-1 increased by 4- and 87-fold, respectively, in the severe model at 7 dpi but only approximately 1.2- and 5.7-fold, respectively, in the mild model. Analysis of the levels of IL-1β, IL-2, IL-4, and IL-10 (Figure S2) revealed no significant difference or obvious sudden induction between the severe and mild mice. Taken together, our data suggest that cytokines IL-5 and IL-13 are likely the main contributors to early inflammatory responses and that the cytokines and chemokines IL-6, MCP-1, CCL5/RANTES, TNF-α, and IFN-γ likely play important roles in the immunopathological lesions observed in the severe pathogenesis of EV71 infection.

## 4. Discussion

Since its first identification in 1969, EV71 has been regarded as a serious neurological threat globally, particularly in the Asia-Pacific region [5,14,15,40,41,42,43]. Severe cases of EV71 infection manifest most frequently as neural syndromes in infants and young children. EV71 outbreaks resulted in neurological diseases such as brainstem encephalitis, aseptic meningitis, motor neuron death, and complications, including neurogenic pulmonary edema and even death [3]. After the eradication of poliovirus, EV71 has been regarded as the most vital neurotropic enterovirus [1,44]. Histological evidence has indicated that the tissue lesion in EV71 encephalitis is due to viral neurotropism [11]. Several studies have demonstrated that EV71 infects target cells, especially human neural cells in vitro, and that apoptosis contributes to neurovirulence [45,46,47]. Experimental EV71 infections of suckling mice have demonstrated that EV71 results in neuronal necrosis and apoptosis in the CNS [23,25]. Experimental studies play a vital role in the elucidation of the mechanisms underlying EV71-related pathogenesis and its fatal prognosis. However, neurovirulence in mouse neurons induced by EV71 infection has been largely unexamined, and mouse models of severe and mild EV71 infection have not been established. In our study, we analyzed the neurovirulence of EV71 strains isolated in China in human and mouse neural cells, respectively. We determined that EV71 strains are neurotropic in human neural cells. In addition, we established mouse models of severe and mild EV71 infection using mild and severe clinical EV71 strains and comprehensively analyzed pathological characteristics, viral replication in tissues, and dynamic changes in serum cytokine and chemokine levels. Our data indicate that these elevated cytokines and chemokines, such as IL-5, IL-13, IL-6, MCP-1, and CCL5/RANTES, may have potential value as severe prognostic markers in mouse models. Therefore, our study provides insights into the neurovirulence of EV71 and potential predicting factors in serum. 

Previous phylogenetic analyses of the complete EV71 genome have demonstrated that the most prominent EV71 strains circulating in China belong to subgenotype C4 [48], which has been proposed as a new “genotype D” [16,17]. In this study, we examined nine EV71 strains obtained from patients diagnosed as fatal, severe, or mild cases of EV71 during the 2010–2012 period in Hunan, Ningxia, and Guangdong provinces by phylogenetic tree analysis, which revealed that all nine strains belong to the C4 subgenotype (proposed as genotype D) (Figure 5). This result also suggests that the strains of EV71 circulating in China in 2010–2012 were related to EV71 subgenotype C4 (proposed as genotype D). Therefore, we compared the infectivity of these EV71 strains in human neuroblastoma SK-N-SH cells and mouse neuroblastoma N2a cells. Our data demonstrated that the EV71 strains isolated from fatal, severe, or mild EV71 patients could infect and replicate in human neural cells, and ultrastructural observation confirmed that the fatal strain was replicated in SK-N-SH cells (Figure 3). These results reveal that the genotype might not be predictive of severity of disease. Previous mouse model studies revealed EV71 genome variations in the strain causing severe disease and showed that when an avirulent strain had that variation inserted, there was increased mortality and clinical disease as well as increased virus in a number of tissues [49]. However, our results reveal that the differences in clinical prognosis of EV71 might not be due to strains. Other potential factors may determine the clinical outcome of HFMD patients with EV71 infection. The infectivity of EV71 to children and clinical manifestations may differ from the strains, exposure dose of virus, the physical condition, age of the children, and the sanitary and medical conditions. Thus, we use two clinical strains in the animal model experiment to mimic the severe and mild clinical manifestations and fit the other uncertain factors. Therefore, these mouse models of severe and mild EV71 infection may be valuable disease models for understanding the neurological pathogenesis of fatal EV71 infection and may be of diagnostic and therapeutic value for EV71 infection. We next investigated whether these EV71 strains could infect mouse neuroblastoma N2a cells. Two of the nine EV71 strains caused CPEs in N2a cells and underwent viral replication. This result indicates that some EV71 clinical strains may not suitable for EV71 infection in mouse neural cells. Previous mouse model studies revealed that EV71 strains cause massive lesions in skeletal muscle and the CNS, eventually leading to death due to neurological lesions [23,25]. However, these models are not convenient for comparing severe and mild EV71-mediated pathogenesis. Our investigation of the infectivity of these non-mouse-adapted EV71 strains in mice demonstrated that only two of the nine clinical strains (NX10-147 and NX10-36) could infect suckling mice, which exhibited hind limb paralysis. Unexpectedly, the strains that infected mice differed from those that infected mouse neural cells in vitro. Our data indicated that the non-mouse-adapted EV71 strain NX10-36 was applicable to both in vivo and in vitro studies. Furthermore, mice inoculated i.p. with 10^6.0^ TCID50 of NX10-36 died of severe EV71 infection lesions (data not shown). In the present study, 9-day-old suckling mice infected i.p. with 10^4.5^ TCID50 of two clinical EV71 strains exhibited severe and mild clinical manifestations, mimicking the clinical prognostic development of EV71 infection in humans. Although our results indicated obvious differences between clinical manifestations and survival rates of the severe and mild mouse models, there was no obvious difference in the viral loads and pathological lesions between the models, strongly indicating a role of other crucial factors in the development of severe EV71 infection. Therefore, we quantitatively analyzed cytokines and chemokines in the sera of the severe and mild mouse models.

The excessive release of cytokines resulted in a systemic inflammatory response, which has explained the pathogenesis of EV71-associated fatality [31]. Previous studies have reported a correlation of proinflammatory cytokines and chemokines with the clinical severity and outcome of EV71 infection [31,32,33,36]. Similar to previous studies on the role of cytokines in clinical severity, we also observed a cytokine storm during the early period of EV71 infection in the severe model. Strikingly, IL-5 was the first detectable cytokine at 6 hpi during the cytokine storm. IL-5 is essential for eosinophil survival in humans and B cell development in mice, and it plays a detrimental role in allergic diseases and parasite infection [50]. IL-5 levels in the sera of EV71 patients with encephalitis have been demonstrated to be an accurate prognostic marker of death [51]. We also observed elevated IL-5 levels in response to severe experimental EV71 infection, suggesting that IL-5 may be a potential prognostic marker of EV71 infection in both humans and mice. IL-6 and IL-13 levels are remarkably higher in patients with CNS infections and pulmonary edema with fatal outcomes than in uncomplicated patients [31,34]. A mouse model study demonstrated that high level of IL-6 might cause tissue lesion and immunopathological responses [52]. Consistent with previous studies, we also observed an increase in IL-6 levels in the severe model at the early stage of infection. The results from this experimental study also suggest that a profile of elevated cytokine and chemokine levels, including IL-5, IL-13, IL-6, MCP-1, and CCL5/RANTES, may be a predictor of fatal outcomes. 

In conclusion, EV71 strains obtained from patients diagnosed as mild, severe, and fatal infections demonstrated infectivity in human neural SK-N-SH cells, suggesting that EV71 virus is strongly neurotropic in humans. Thus, other potential factors, including cytokines and chemokines in the serum, may play an important role in clinically mild, severe, or fatal prognoses. Furthermore, we established the first mouse models of severe and mild EV71 infection. Most importantly, elevated cytokines and chemokines, including IL-5, IL-13, IL-6, MCP-1, and CCL5/RANTES, may have potential value as biomarkers and may contribute to immunopathological lesions. Therefore, these mouse models of severe and mild EV71 infection may be valuable disease models for understanding the neurological pathogenesis of fatal EV71 infection and may be of diagnostic and therapeutic value for EV71 infection.

## Supplementary Materials

The following are available online at http://www.mdpi.com/1999-4915/9/11/351/s1
