# Supplementary material for: Neurotropism In Vitro and Mouse Models of Severe and Mild Infection with Clinical Strains of Enterovirus 71
Source: Viruses. 2017 Nov 20;9(11):351. doi: 10.3390/v9110351 (PMC5707558; doi:10.3390/v9110351)
Supplement: Supplementary file 1 [file viruses-09-00351-s001.pdf]

Neurotropism in vitro and mouse models of severe and mild infection with clinical strains of enterovirus 71

Pin Yu, Linlin Bao, Lili Xu, Fengdi Li, Qi Lv, Wei Deng, Yanfeng Xu, and Chuan Qin*

Institute of Laboratory Animal Sciences, Chinese Academy of Medical Sciences (CAMS) & Comparative Medicine Center, Peking Union Medical College (PUMC); Key Laboratory of Human Diseases Comparative Medicine, Ministry of Health; Key Laboratory of Human Diseases Animal Model, State Administration of Traditional Chinese Medicine, Beijing 100021, China

*Corresponding author.

Mailing address: Institute of Laboratory Animal Sciences, No. 5 of Pan Jia Yuan Nan Li, Chao Yang District, Beijing, 100021, China

Tel: +86-10-67761942

FAX: +86-10-67761943

E-mail address: qinchuan@pumc.edu.cn

Table S1. EV71 viruses isolated from different regions in China

**EV71 strain**	**GenBank accession number**	**Clinical characteristic**	**Region (province)**	**Year**	**Subgenotype (proposed genotype)**
**Hun11-32**	KJ004552	Fatal	Hunan	2012	C4 (D)
**Hun11-4**	KJ004553	Mild	Hunan	2011	C4 (D)
**Hun12-14**	KJ004554	Fatal	Hunan	2012	C4 (D)
**Hun12-10**	KJ004555	Mild	Hunan	2012	C4 (D)
**NX10-147**	KJ004556	Mild	Ningxia	2010	C4 (D)
**NX10-36**	KJ004557	Severe	Ningxia	2010	C4 (D)
**GD10-7**	KJ004558	Severe	Guangdong	2010	C4 (D)
**GD10-12**	KJ004559	Mild	Guangdong	2010	C4 (D)
**GD10-45**	KJ004560	Fatal	Guangdong	2010	C4 (D)

Table S2. Oligonucleotide primers for the amplification of the EV71 genome

Primer	Region	Position	Sense	Sequences(5'-3')
1F	5' UTR	1-20	Forward	5-TTAAAACAGCCTGTGGGTTG
1R	VP4	923-950	Reverse	ACTTCAGTGGCGCTGCCATTTCAGTGAA
2F	VP4	743-768	Forward	ATGGGTTCGCAAGTGTCTACACAGCG
2R	VP2	1459-1489	Reverse	GTGTGGGCACACTGTTAACTGTGATATTGGG
3F	VP2	1160-1188	Forward	TGGGAGAAATCGTCCAAGGGATGGTACTG
3R	VP1	2708-2736	Reverse	TGTGCCCTTAAGAGGGAGATCTATCTCTC
4F	VP1	2438-2466	Forward	GGAGATAGGGTGGCAGATGTAATTGAAAG
4R	2A	3749-3777	Reverse	CTGTTCCATAGCTTCTTCATCTAACCACA
5F	2A	3311-3339	Forward	ACAGCGATCACCACTCTTGGGAAATTTGG
5R	2C	4344-4361	Reverse	TGGCGTATAGCGGTTGGA
6F	2C	4258-4275	Forward	AAACTTGGAACAATCTGC
6R	3A	5228-5245	Reverse	GACAAGCACCGCTCTATT
7F	3A	5063-5089	Forward	GGTCCACCCAAGTTCAGGCCAATTAGG
7R	3C	5904-5935	Reverse	TTGTTCACTAGCAAAGTAACTCCTTTTGAGGC
8F	3C	5582-5599	Forward	CAAGGAGTCAACCTGGAA
8R	3D	6926-6956	Reverse	AATCAATTGGGAAGGGATAGCTAGCGAGCAC
9F	3D	6596-6624	Forward	CTGCCAATTTTGCTCCCTGGCTCACTCTT
9R	3' UTR	7387-7405	Reverse	GCTATTCTGGTTATAACAA

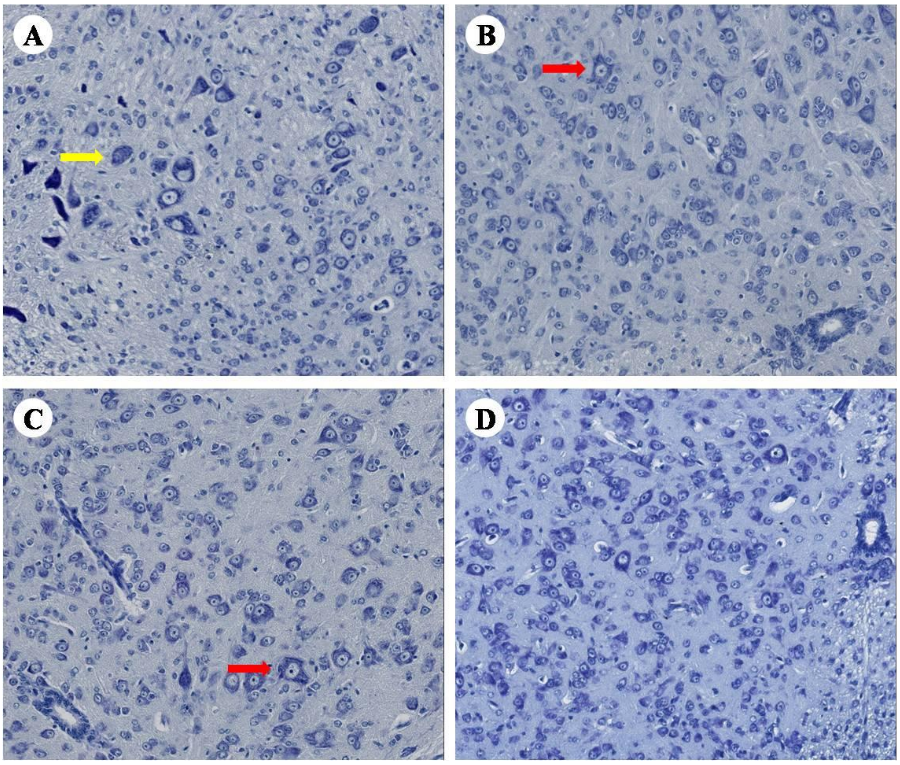

Figure S1. Pathological changes in the central nervous system (CNS) of EV71-infected mice detected by Nissl staining. Nine-day-old mice were inoculated i.p. with $10^{4.5}$ TCID50 of EV71 NX10-147 (shown in A and B) or NX10-36 (shown in C), and histopathological examination of the CNS was performed at 5 dpi by Nissl staining. Pathological lesions in the CNS were observed in infected mice. Nerve cells exhibited a disordered arrangement, and some exhibited a decrease in Nissl bodies in the brainstem (A, yellow arrow) and spinal cord (B and C, red arrow). Normal tissues are shown (D).

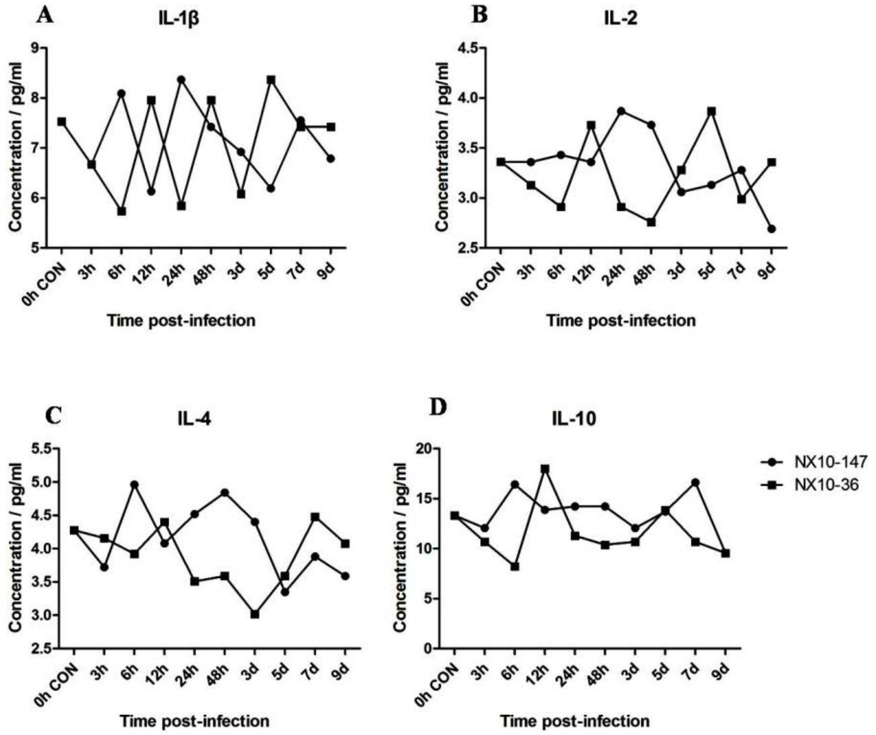

Figure S2. Dynamic detection of inflammatory cytokines and chemokines in the sera of EV71 (NX10-147 and NX10-36)-infected mice sacrificed at 3, 6, and 12 hpi and 1, 2, 3, 5, 7, and 9 dpi. The serum concentrations of cytokines (A) IL-1β, (B) IL-2, (C) IL-4, and (D) IL-10 were analyzed using a cytometric bead array.
